# d-Amino Acids Do Not Inhibit Biofilm Formation in *Staphylococcus aureus*

**DOI:** 10.1371/journal.pone.0117613

**Published:** 2015-02-06

**Authors:** Sourav Sarkar, Marcos M. Pires

**Affiliations:** Department of Chemistry, Lehigh University, Bethlehem, Pennsylvania, United States of America; Ghent University, BELGIUM

## Abstract

Bacteria can either exist in the planktonic (free floating) state or in the biofilm (encased within an organic framework) state. Bacteria biofilms cause industrial concerns and medical complications and there has been a great deal of interest in the discovery of small molecule agents that can inhibit the formation of biofilms or disperse existing structures. Herein we show that, contrary to previously published reports, d-amino acids do not inhibit biofilm formation of *Bacillus subtilis* (*B. subtilis*), *Staphylococcus aureus* (*S. aureus*), and *Staphylococcus epidermis* (*S. epidermis*) at millimolar concentrations. We evaluated a diverse set of natural and unnatural d-amino acids and observed no activity from these compounds in inhibiting biofilm formation.

## Introduction

Most bacteria escape environmental chemical and physical stresses by forming complex matrix encapsulated aggregates known as biofilms [1]. The biofilm matrix consists of a polymeric conglomeration of exo-polysaccharides, proteins and extracellular DNA [2,3]. Biofilm formation is crucial in the lifecycle of bacteria and is tightly regulated with both spatial and temporal control. In bacterial cultures, biofilm develops either at the liquid-air interface (e.g., *B*. *subtilis*) [4] or at the solid-liquid interface (e.g., *S*. *aureus* and *S*. *epidermis*) [4,5,6,7]. With these models in hand, great strides have been made in terms of obtaining key insight into the steps that go into the formation of biofilms, the identities of the macromolecules contained within these matrices, and the phenotypic changes that occur during this process.

Microbial biofilms are of significant environmental and biomedical importance [8,9]. Formation of biofilms can have severe detrimental and life-threatening impact in clinical settings [10]. For example, bacterial biofilms frequently develop on surgical implants, which may lead to deteriorating performance and further complications [8,9,11]. Most significantly, over 80 percent of all incidences of microbial infections are related to biofilms [11]. Biofilm–associated bacterial cells are inherently more tolerant to antibiotic treatment and can lie dormant to resist the actions of the antimicrobial agents [11,12]. The development of effective small molecule agents that can disrupt biofilm formation and disperse existing biofilm is of prime importance [13,14,15,16,17]. Recently, the field was invigorated by the demonstration that unnatural D-amino acids can effectively inhibit and disperse bacterial biofilms [4,5,18]. Media from aged disrupted biofilm cultures were shown to contain significant levels of D-amino acids and the same exhausted media was sufficient to prevent biofilm formation in fresh cultures. Importantly, the enantiomeric L-amino acid counterparts failed to show any activity in biofilm disruption and disassembly, highlighting the specificity and hinting at a mechanism of inhibition. It was hypothesized that D-amino acid inhibition of biofilm formation proceeded *via* the insertion of the unnatural D-amino acids in place of the terminal D-alanine in the stem peptide of the bacterial peptidoglycan [19]. While single amino acid treatment proved to be effective, synergistic effects were also observed with the concurrent incubation of three unnatural D-amino acids.

The promising anti-biofilm activities reported for D-amino acids led us to investigate a large series of unnatural D-amino acids in search of more potent D-amino acid agents. We hypothesized that by exploring a larger and more diverse chemical space for the side chain of the D-amino acid, we would discover potent inhibitors of bacterial biofilm. Instead, we found that, contrary to published reports, none of the unnatural D-amino acids we evaluated displayed specific inhibition of biofilm formation against strains of *B*. *subtilis*, *S*. *epidermis*, and *S*. *aureus* at millimolar concentrations.

## Materials and Methods

### General Methods

All D-amino acids were purchased from either ChemImpex or PepTech (98%+ purity). NaCl, MnCl_2_ and glycerol were purchase from Fischer. Luria broth (LB) and Tryptic soy broth (TSB) were purchase from Sigma and BD respectively. Costar 3628 96-well and Costar 3337 24-well sterile polystyrene, flat bottom tissue culture treated plates with low evaporation lid were purchased from corning. Absorbance was recorded on a Tecan Infinite F200 plate reader at 595 nm. Images were recorded on a Canon Powershot ELPH 100HS digital camera.

### Preparation of D-amino acid Stock Solution

Individual stock solutions of 100 mM of D-amino acids were prepared by dissolving appropriate amounts of each D-amino acids into 0.2 M NaOH.

### *S*. *aureus* (SC01 and ATTC 12228) biofilm assay (with 1 mM of D-amino acids)

*S*. *aureus* SC01 were grown in LB overnight at 37°C with shaking and diluted 1:100 in TSB medium supplemented with NaCl (3%), glucose (0.5%), appropriate D-amino acid stocks in 0.2 M NaOH (1 mM), HCl (4 mM) and PBS (0.5X). This bacterial culture was then transferred (200 μL) into 96-well plates and incubated either 24 h or 48 h with low evaporation lid at 37°C without shaking and covered with aluminum foil to protect from light. The supernatant were discarded and the plates were washed twice with PBS (1X, 200 μL) and dried at 65°C for 1 h. The plates were then cooled to room temperature and stained with 1% crystal violet for 10 min. The plates were then washed twice with double distilled water (200 μL) and dried overnight. The crystal violet stained biofilms were dissolved by adding 95% ethanol (200 μL) to each well and shaking for 2 h. The contents were diluted 20 times and then the absorbance was recorded at 595 nm.

### *S*. *aureus* SC01 biofilm assay (with 5 mM of D-amino acids)

*S*. *aureus* SC01 were grown in LB overnight at 37°C with shaking and diluted 1:100 in TSB medium supplemented with NaCl (3%), glucose (0.5%), appropriate D-amino acid (D-Trp, D-Tyr, D-Tyr/ D-Pro/ D-Phe mix) stocks in 0.2 M NaOH (5 mM), HCl (20 mM) and PBS (0.5X). For untreated cells, double distilled water was added instead of the D-amino acids. These bacterial cultures were then transferred (800 μL) into a 24-well plate and incubated for 24 h with a low evaporation lid at 37°C without shaking. The supernatant was discarded and the plate was washed twice with PBS (1X, 1 mL) and dried at 65°C for 1 h. The plate was then allowed to cool to room temperature and stained with 1% crystal violet (500 μL) for 10 min. The plate was then washed twice with double distilled water (1 mL) and dried overnight. Photographs of the stained wells were recorded at this time. The crystal violet stained biofilms were dissolved by adding 95% ethanol (500 μL) to each well and shaking for 2 h. The contents were diluted 20 times and then the absorbance was recorded at 595 nm.

### *S*. *epidermis* ATTC 12228 biofilm assay (with 1 mM of D-amino acids)

*S*. *epidermis* ATCC 12228 were grown in LB overnight at 37°C with shaking and diluted 1:100 in TSB medium supplemented with NaCl (3%), glucose (0.5%), appropriate D-amino acid stocks in 0.2 M NaOH (1 mM), HCl (4 mM) and PBS (0.5X). The bacterial cultures was then transferred (200 μL) into 96-well plates and incubated for 48 h with a low evaporation lid at 37°C without shaking and covered with aluminum foil to protect from light. The supernatant was discarded and the plates were washed twice with PBS (1X, 200 μL) and dried at 65°C for 1 h. The plated were then allowed to cool to room temperature and stained with 1% crystal violet for 10 min. The plates were then washed twice with double distilled water (200 μL) and dried overnight. The crystal violet stained biofilms were dissolved by adding 33% glacial acetic acid (200 μL) to each well and shaking for 2 h. The contents were diluted 20 times and then the absorbance was recorded at 595 nm.

### *B*. *subtilis* 3610 biofilm assay (with 1 mM of D-amino acids)

*B*. *subtilis* 3610 were grown in LB overnight at 37°C with shaking and diluted 1:1000 in LB supplemented with glycerol (3% v/v), MnCl_2_ (0.1 mM), appropriate D-amino acid stocks in 0.2 M NaOH (1 mM), HCl (4 mM) and PBS (0.5X). These bacterial cultures were then transferred (200 μL) into 96-well plates and incubated for 72 h with a low evaporation lid at room temperature without shaking and covered with aluminum foil to protect from light. The biofilms formation was analyzed by recording images.

## Results and Discussion

**Fig. 1:**
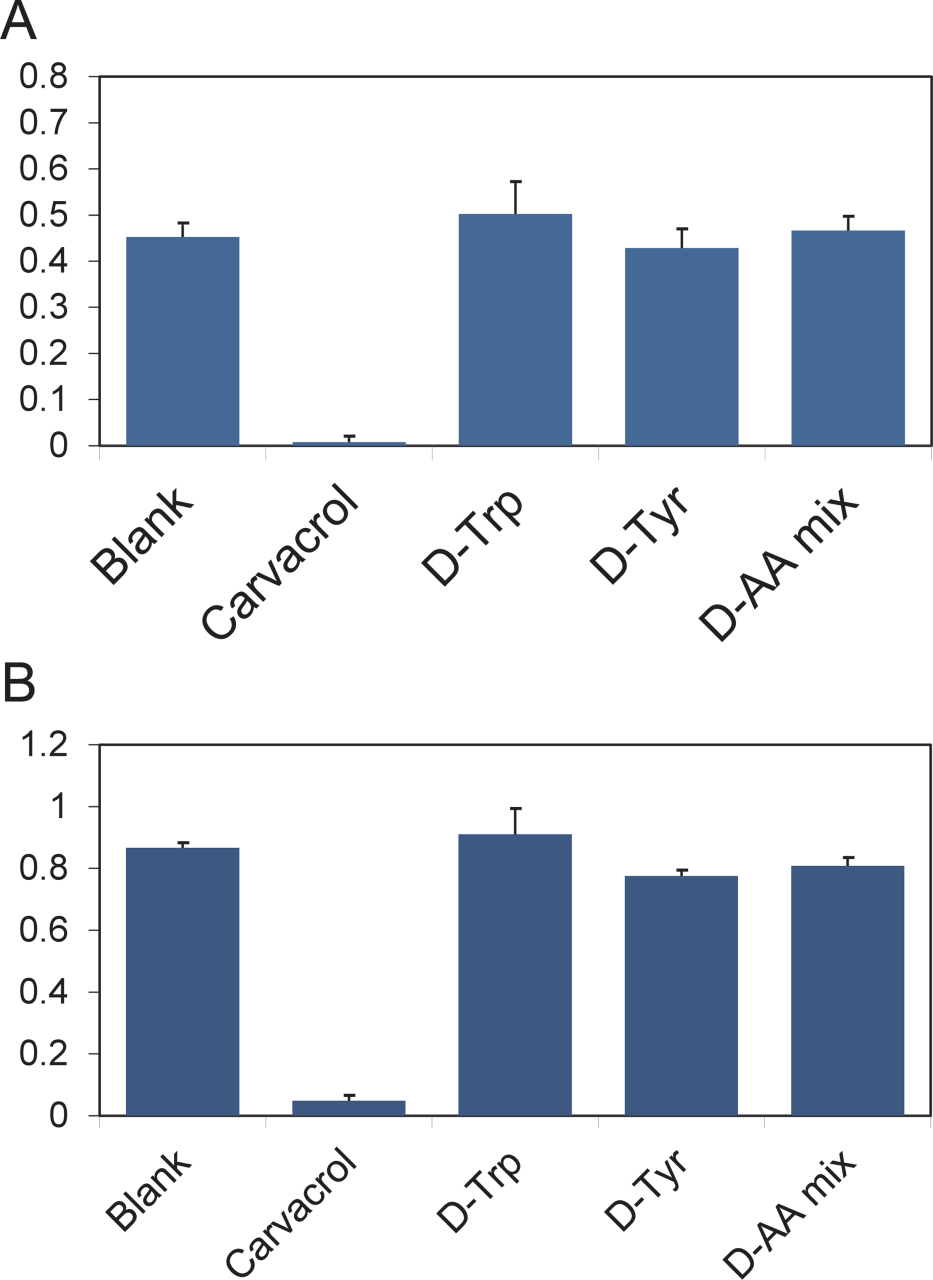
Control compounds effect on biofilm formation. *S*. *aureus* (SC01) biofilm formation was evaluated after 24 h (A) and 48 h (B) in the presence of D-amino acids previously reported as inhibitors at a concentration of 1 mM or the positive control carvacrol (2 mM). The absorbance was recorded at 595 nm following crystal violet staining. All points are significantly different from the positive control carvacrol (****p<0.0001) relative to positive control, unpaired t-test.).

**Fig. 2:**
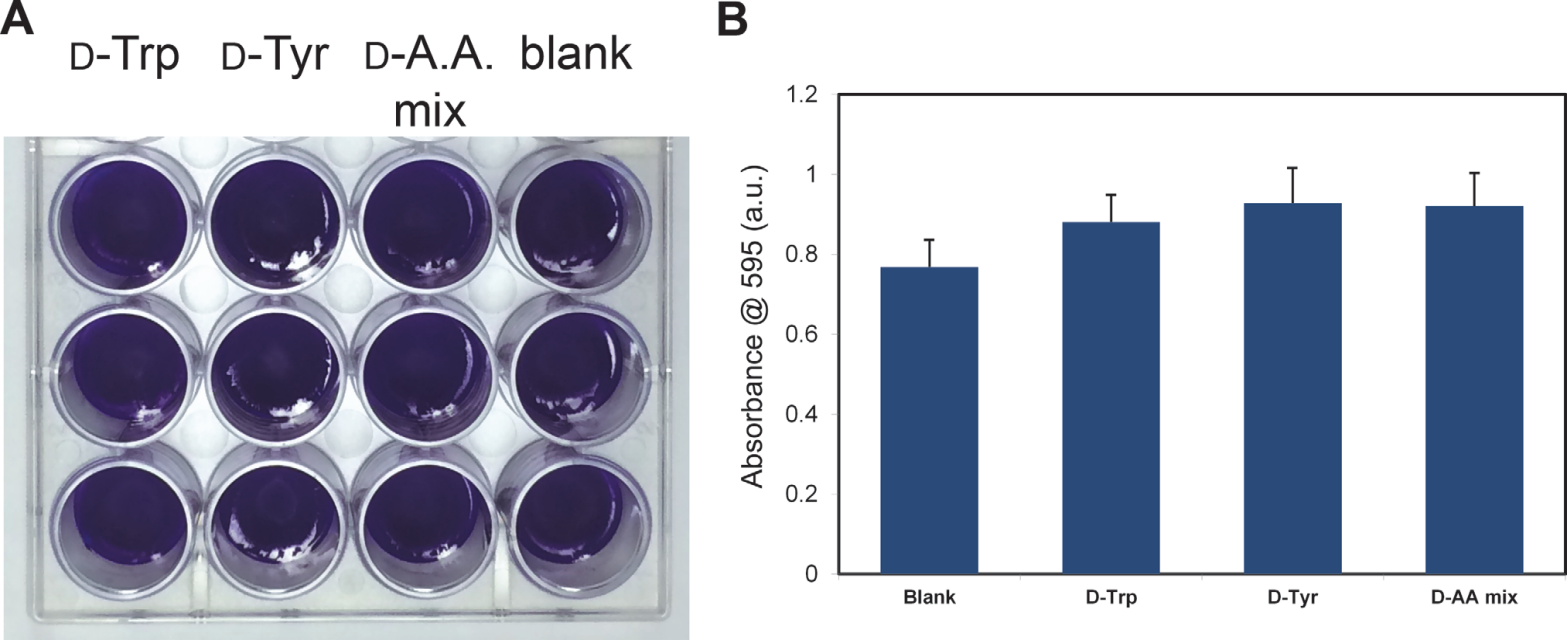
Effect of increased concentration of control compounds. *S*. *aureus* (SC01) biofilm formation was evaluated visually (A) and spectroscopically (B) after 24 h in the presence of D-tyrosine, D-tryptophan, and an equimolar mixture of D-tyrosine/D-proline/D-phenylalanine at a concentration of 5 mM.

In order to evaluate the effect of D-amino acids on biofilm formation we initially used *S*. *aureus* (SC01 strain), the same strain that had been previously shown to be sensitive to D-amino acids [5]. In the initial report, D-tyrosine was reported to fully inhibit biofilm formation of *S*. *aureus* at 50 μM and the mixture of D-leucine/D-methionine/D-tyrosine/D-tryptophan at 15 nM each [4]. In a later report, the potency of D-tyrosine was revised. They found that D-phenylalanine, D-proline, and D-tyrosine were individually able to fully inhibit biofilm formation at 500 μM [5]. The combination of these three D-amino acids led to significant impairment at 10 μM and full inhibition at 100 μM [5]. Initially, we set out to reproduce these results as control compounds in inhibiting the formation of *S*. *aureus* biofilm as previously reported. We observed that D-tyrosine, D-tryptophan, and the equimolar mixture of D-tyrosine/D-proline/D-phenylalanine failed to inhibit biofilm formation at 1 mM (**Fig. 1**). Visual inspection of the biofilm confirmed that none of the compounds disrupted biofilm formation. Next, we doubled the incubation time to 48 hours to explore the possible inhibition by D-amino acids at longer incubation times. Clearly, the biofilms became denser at the 48-hour period as seen by the higher crystal violet-associated absorbance for all the compounds evaluated. Next, we evaluated the ability of D-amino acids to inhibit bacterial biofilm formation at 5 mM, which is ten times higher than the reported revised concentration for full inhibition and a hundred times higher than the initially reported concentration. For this series, only the reported biofilm inhibitors (D-tyrosine, D-tryptophan, and the equimolar mixture of D-tyrosine/D-proline/D-phenylalanine) were assessed at 5 mM. Following a 24 hour incubation period, there was no biofilm inhibition based on visual inspection and no significant inhibition was observed following staining (**Fig. 2**). Our data indicates that the D-amino acids evaluated herein show no activity in modulating the formation of *S*. *aureus* biofilm.

**Fig. 3:**
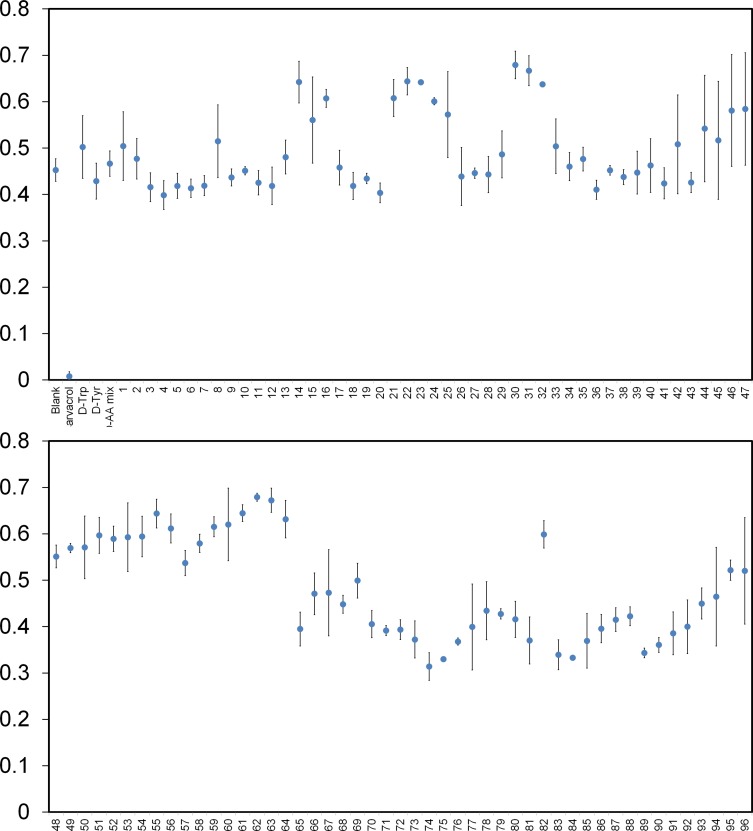
Diverse set of D-amino acids effect on biofilm formation. *S*. *aureus* (SC01) biofilm formation was evaluated after 24 h in the presence of specified unnatural D-amino acids at a concentration of 1 mM or the positive control carvacrol (2 mM). The absorbance was recorded at 595 nm following crystal violet staining. All points are significantly different from the positive control carvacrol (****p<0.0001 relative to positive control, unpaired t-test.).

**Fig. 4:**
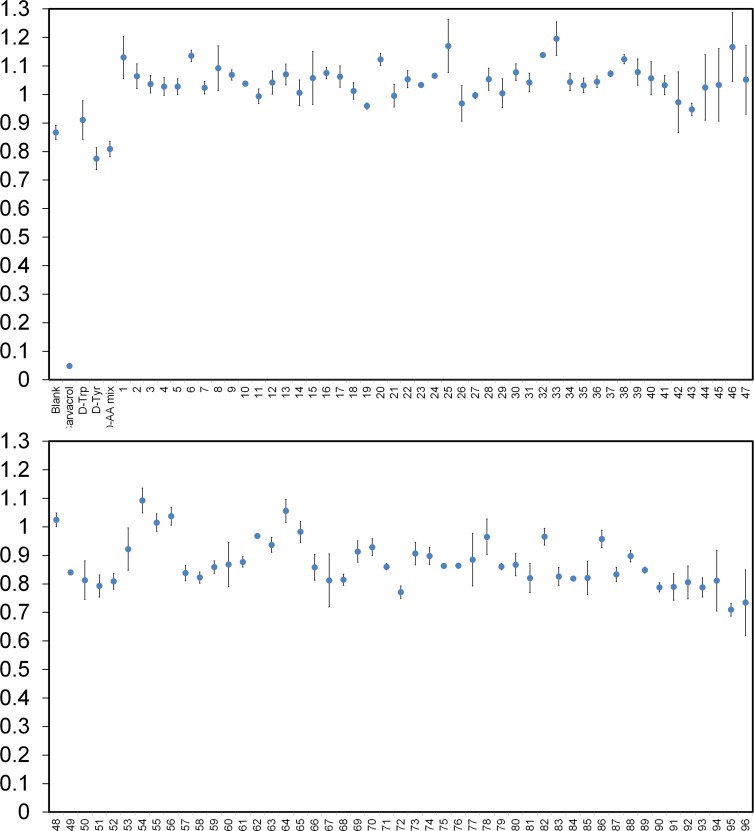
Diverse set of D-amino acids effect on biofilm formation. *S*. *aureus* (SC01) biofilm formation was evaluated after 48 h in the presence of specified unnatural D-amino acids at a concentration of 1 mM or the positive control carvacrol (2 mM). The absorbance was recorded at 595 nm following crystal violet staining. All points are significantly different from the positive control carvacrol (****p<0.0001 relative to positive control, unpaired t-test.).

Next, we compiled a diverse library of 96 unnatural D-amino acids, including D-tyrosine as the reference positive control compound (compounds **1**–**96, Table A** in **S1 File**). On average, the compounds were approximately 203 Da in molecular weight and all contained the free termini and D-stereochemistry. We had predicted that aromatic side chains on D-amino acids (similar to D-tyrosine) derivatized with either electron donating or electron withdrawing groups would help us to determine the appropriate structural elements required for effective biofilm disruption. Therefore, a large number of the D-amino acids included in the library contained elements that resemble D-tyrosine such as hydrogen bond donors and acceptors (e.g., nitrogen atoms). Additionally, a number of derivatives contained halogenated aromatic side-chains. The placement of these heteroatoms were diversified by their positioning along the aromatic ring (ortho, para, meta positions). We also included several non-aromatic side chains within our library of D-amino acids to probe a diversified chemical space. Following a 24-hour incubation period to induce *S*. *aureus* biofilm formation, no inhibition was observed for the entire set of compounds that were screened at 1 mM (Fig. 3 and see additional figures in **S1 File** for larger version of the same data). As a positive control, we included the established *S*. *aureus* biofilm inhibitor carvacrol (**Fig. C** in **S1 File**).[20] Based on the stringent cut-off of 5 standard deviations from the mean, none of the D-amino acids or their combinations displayed biofilm inhibition. Extending the incubation to 48 h led to overall increased signals but did not change the results (Fig. 4). We believe that this is the largest reported screen of D-amino acids to date against biofilm formation and it is consistent with the negative results observed for the control compounds. From this data, we conclude that D-amino acids do not inhibit *S*. *aureus* biofilm formation at the concentrations tested.

Next, we were interested in exploring the effect of D-amino acids on the biofilm formation of a different strain of *S*. *aureus*. In this screen, the same set of unnatural D-amino acids was evaluated along with the previously reported positive controls against *S*. *aureus* (ATCC 25923). This particular strain of *S*. *aureus* is widely used as a model biofilm-forming strain and should complement our initial screen using SC01 [19,21,22]. Each unnatural D-amino acid was evaluated at 1 mM for a 48-hour incubation period. None of the compounds from the library were found to be effective at inhibiting biofilm formation (see **Fig. D** in **S1 File**). Once again, D-tyrosine and D-tryptophan displayed no significant inhibition even at the elevated concentration of 1 mM. Together, we concluded that all the unnatural D-amino acids evaluated show no activity in inhibiting *S*. *aureus* biofilm formation in two different strains.

In further evaluating our panel of unnatural D-amino acids, we explored the effects of our compounds with another biofilm forming microorganism. *S*. *epidermis* is a pathogen that frequently causes complications with implants due to their ability to form biofilms at the interface with human tissue [23,24,25,26]. We investigated the effect of our panel of unnatural D-amino acids at 1 mM on the biofilm development of *S*. *epidermis* (ATCC 12228) strain. Similar to our results with *S*. *aureus*, we observe no inhibition of biofilm development with any of the unnatural D-amino acids in our library after 48 hours of incubation in biofilm medium (See **Fig. E** in **S1 File**). Of note, neither D-tyrosine nor D-tryptophan led to a decrease in biofilm formation based on visual inspection and crystal violet staining.

Finally, we were interested in determining the effect of our unnatural D-amino acid library on biofilm formation for the widely used model organism *B*. *subtilis*. *B*. *subtilis* are capable of forming distinct biofilms at the air-liquid interface in liquid culture [27,28,29,30]. We evaluated the effect of each unnatural D-amino acid against *B*. *subtilis* (3610 strain) at 1 mM. Previously, it had been demonstrated that various D-amino acids were potent inhibitors of *B*. *subtilis* biofilm formation, with D-tyrosine showing low micromolar activity [4]. Recently, the same group discovered that the inhibitory effects by D-tyrosine were an artifact due to a mutation in the gene encoding D-tyrosyl-*t*RNA deacylase [18]. Upon repair of the gene, *B*. *subtilis* were no longer sensitive to the actions of D-tyrosine, a clear indication that biofilm inhibition was being mediated by a mechanism distinct from the originally proposed mechanism. In agreement with their findings, we observe a similar trend for the D-amino acids within our panel of compounds upon incubation for three days at concentrations of 1 mM (see **Fig. F** in **S1 File**). As expected, we observed that D-tyrosine and the D-tyrosine/D-proline/D-phenylalanine mixture inhibited biofilm formation in *B*. *subtilis* after three days whereas D-tryptophan inhibited biofilm formation to a moderate extent. The sensitivity to D-tryptophan is consistent with other organisms whose D-tyrosyl-*t*RNA deacylase activity has been inactivated [18,31]. It should be noted that for some of the compounds, the solution became orange during the incubation period with the cells. The coloration was a result of the inherent absorbance properties of the D-amino acid variants. The same color was also observed in the absence of bacterial cells. For example, the treatment of mutated *Escherichia coli* with D-tryptophan led to toxicity at levels that were absent in the wildtype cells [32]. The finding that *B*. *subtilis* are not sensitive to any of the molecules within our panel of D-amino acids suggests that this may not be a viable strategy for the inhibition and dispersion of biofilm of this organism.

## Conclusion

The complications brought on by the development of bacterial biofilms can be immense. In industrial settings, the removal of biofilms requires remediation steps that are often highly costly and procedurally difficult. In medical settings, bacterial biofilms require interventions that can be life-threatening. There is a clear unmet need for the prevention of biofilm formation and the dispersion of existing biofilm. The use of anti-biofilm small molecules could potentially provide a strategy to combat biofilm formation. However, it has been difficult to discover compounds that are potent anti-biofilm agents. It was recently reported that unnatural D-amino acids could inhibit the formation and disperse existing biofilms for three distinct types of bacteria. In contrast to the published reports, we determined that the reported active D-amino acids (D-tyrosine, D-tryptophan, and combinations of D-amino acids) failed to inhibit biofilm formation in three different types of bacteria: *S*. *aureus*, *S*. *epidermis*, and *B*. *subtilis*. Likewise, none of the D-amino acids from our panel of compounds displayed anti-biofilm activity.

## Supporting Information

S1 File This file contains Table A and Figs. A-F. Table A, Structures of unnatural D-amino acids evaluated. The full structure of the diverse set of D-amino acids evaluated are shown below. Fig. A, *S*. *aureus* (SC01) biofilm formation was evaluated after 24 h in the presence of specified D-amino acids at a concentration of 1 mM. The absorbance was recorded at 595 nm following crystal violet staining. Fig. B, *S*. *aureus* (SC01) biofilm formation was evaluated after 48 h in the presence of specified D-amino acids at a concentration of 1 mM. The absorbance was recorded at 595 nm following crystal violet staining. Fig. C, *S*. *aureus* (SC01) biofilm formation was evaluated after 24 h in the absence of cells, in the presence of cells, and in the presence of the positive control carvacrol (2 mM). The images were taken following crystal violet staining. Fig. D, *S*. *aureus* (ATCC 25923) biofilm formation was evaluated after 48 h in the presence of specified D-amino acids at a concentration of 1 mM. The absorbance was recorded at 595 nm following crystal violet staining. Fig. E, *S*. *epidermis* (ATCC 12228) biofilm formation was evaluated after 48 h in the presence of specified D-amino acids at a concentration of 1 mM. The absorbance was recorded at 595 nm following crystal violet staining. Fig. F, *B*. *subtilis* (3610 strain) biofilm formation was evaluated after 3 d in the presence of controls (A) and specified D-amino acids from our panel at a concentration of 1 mM.
(DOCX)
