# Supplementary material for: d-Amino Acids Do Not Inhibit Biofilm Formation in Staphylococcus aureus
Source: PLoS One. 2015 Feb 6;10(2):e0117613. doi: 10.1371/journal.pone.0117613 (PMC4319739; doi:10.1371/journal.pone.0117613)

**Supporting Information**

**D-Amino Acids Do Not Inhibit Biofilm Formation in *Staphylococcus aureus***

Sourav Sarkar & Marcos M. Pires*

Department of Chemistry, Lehigh University, Bethlehem, Pennsylvania 18015, United States.

Correspondence and requests for materials should be addressed to M.P. (email: map311@lehigh.edu)

**Table A.** Structures of unnatural D-amino acids evaluated.


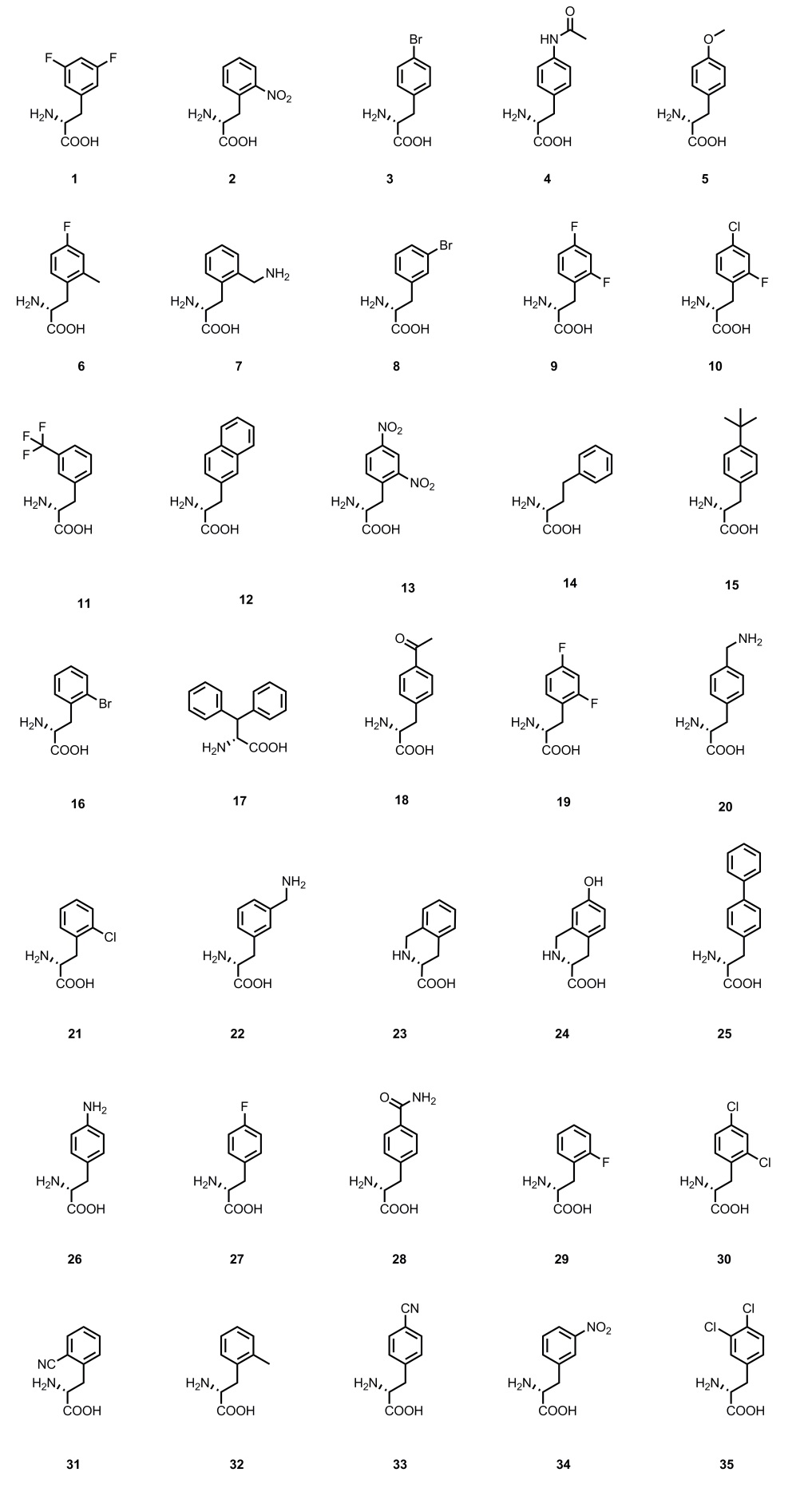


**Table A (continued).**


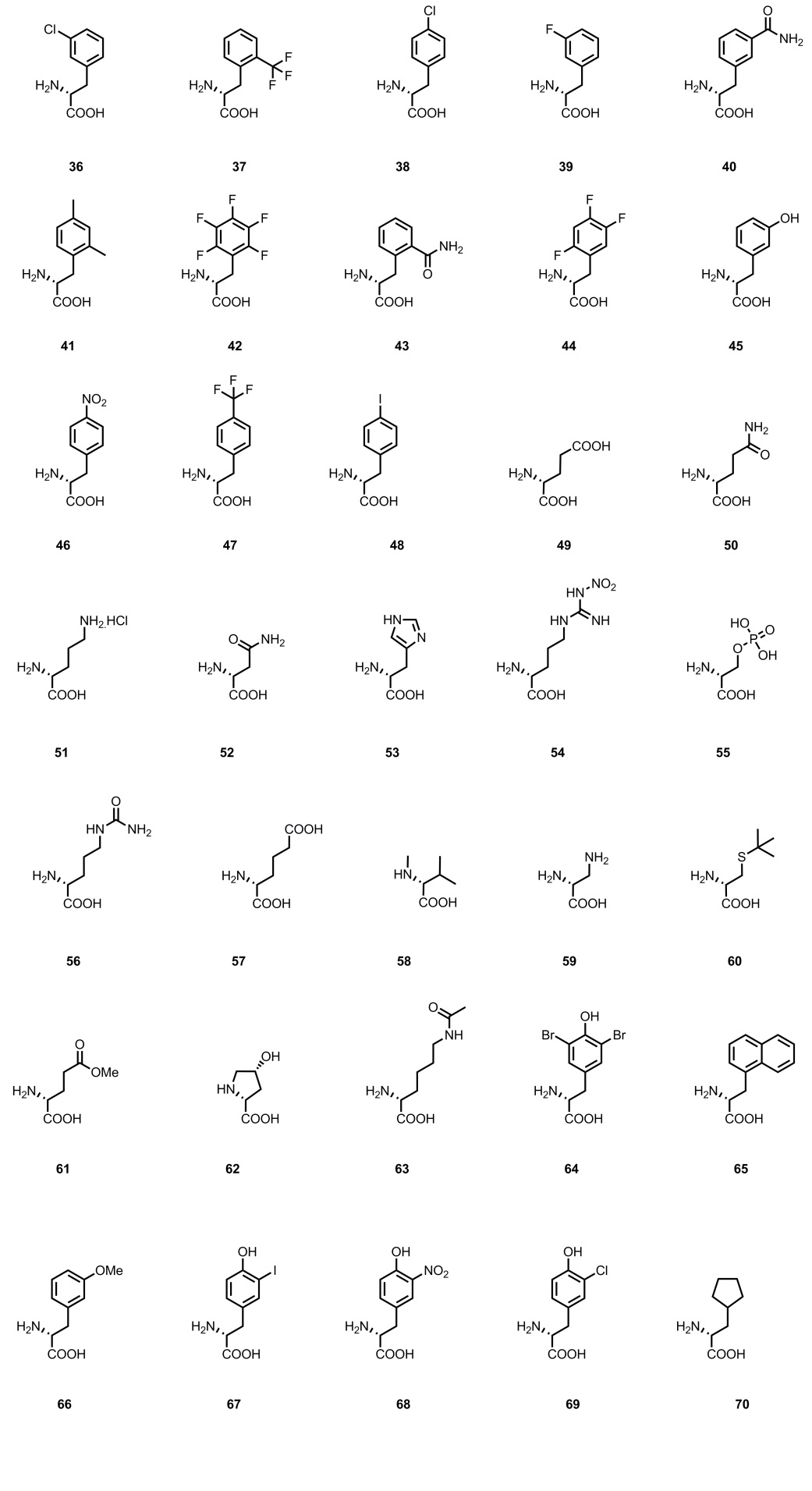


**Table A (continued).**


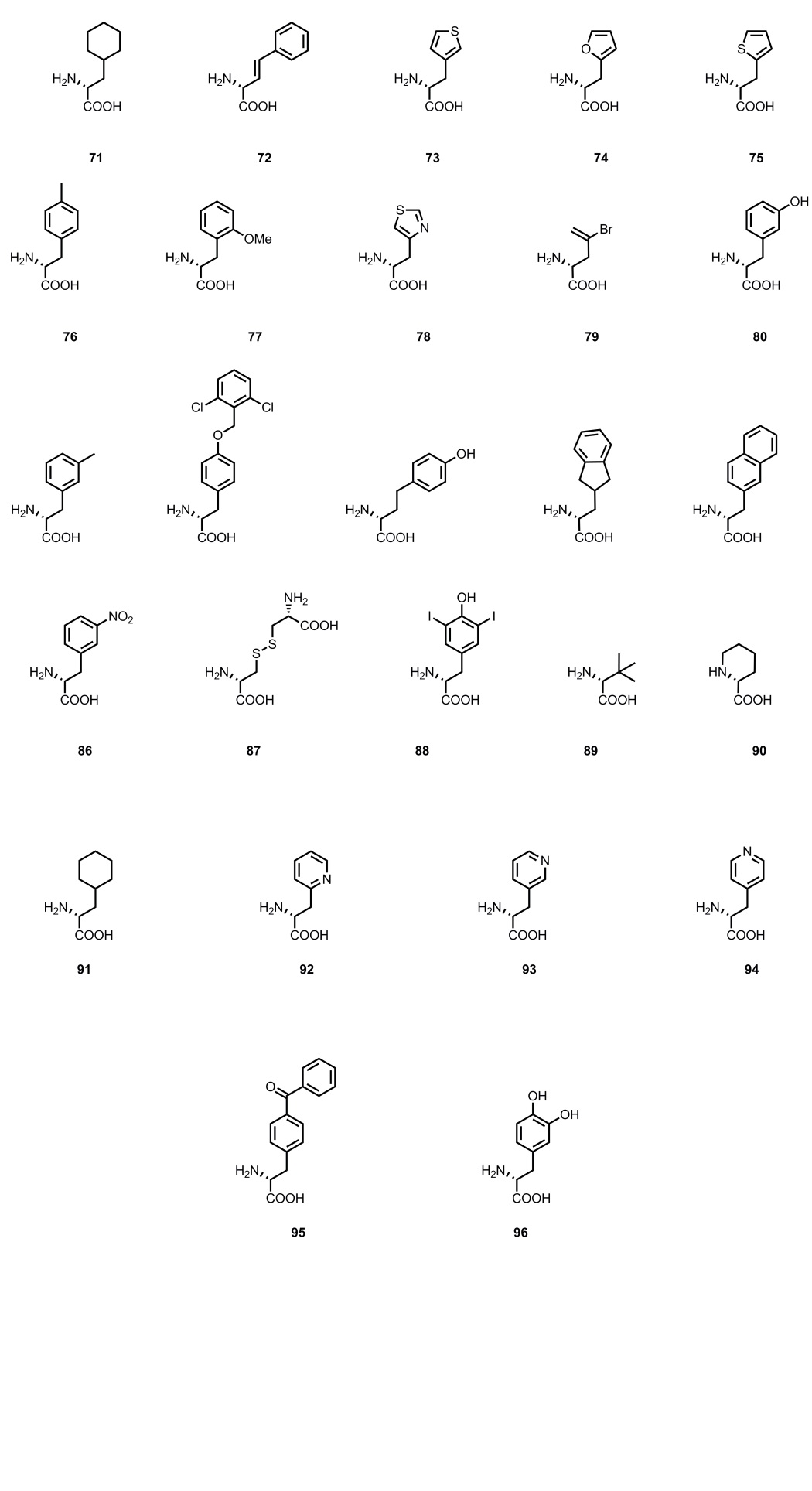


**Figure A.** *S. aureus* (SC01) biofilm formation was evaluated after 24 h in the presence of specified D-amino acids at a concentration of 1 mM. The absorbance was recorded at 595 nm following crystal violet staining.


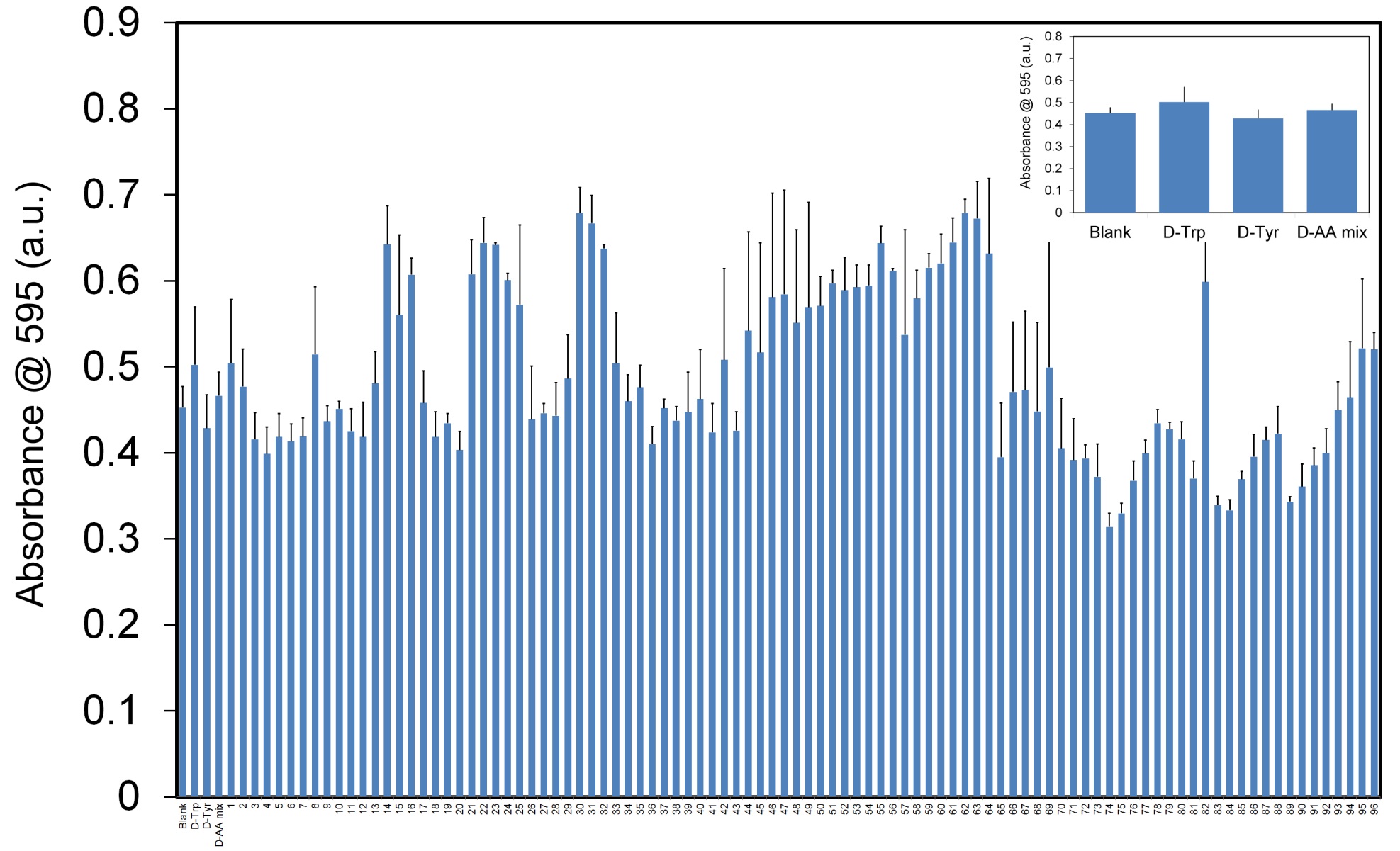


**Figure B.** *S. aureus* (SC01) biofilm formation was evaluated after 48 h in the presence of specified D-amino acids at a concentration of 1 mM. The absorbance was recorded at 595 nm following crystal violet staining.


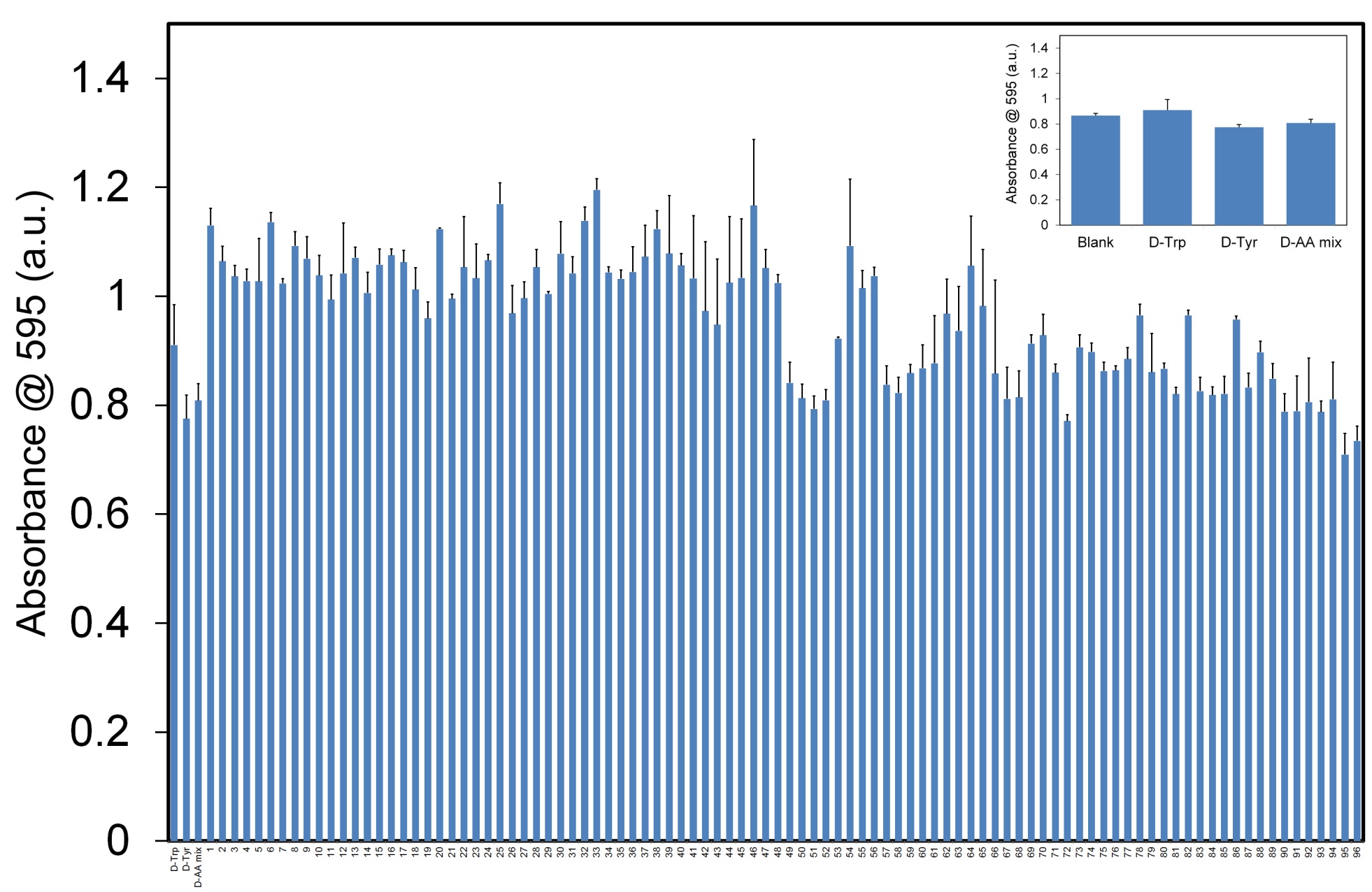


**Figure C.** *S. aureus* (SC01) biofilm formation was evaluated after 24 h in the absence of cells, in the presence of cells, and in the presence of the positive control carvacrol (2 mM). The images were taken following crystal violet staining.

**
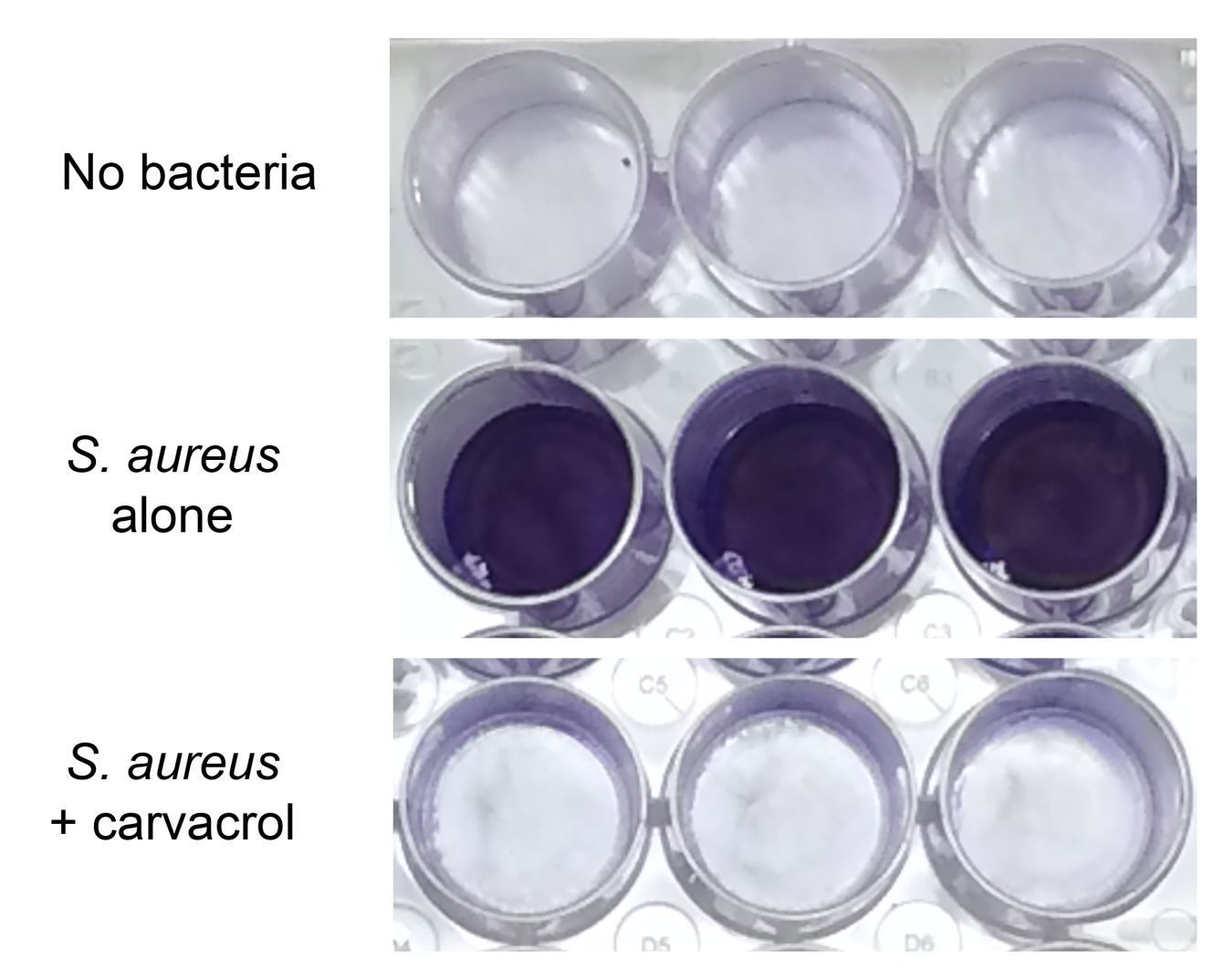
**

**Figure D.** *S. aureus* (ATCC 25923) biofilm formation was evaluated after 48 h in the presence of specified D-amino acids at a concentration of 1 mM. The absorbance was recorded at 595 nm following crystal violet staining.


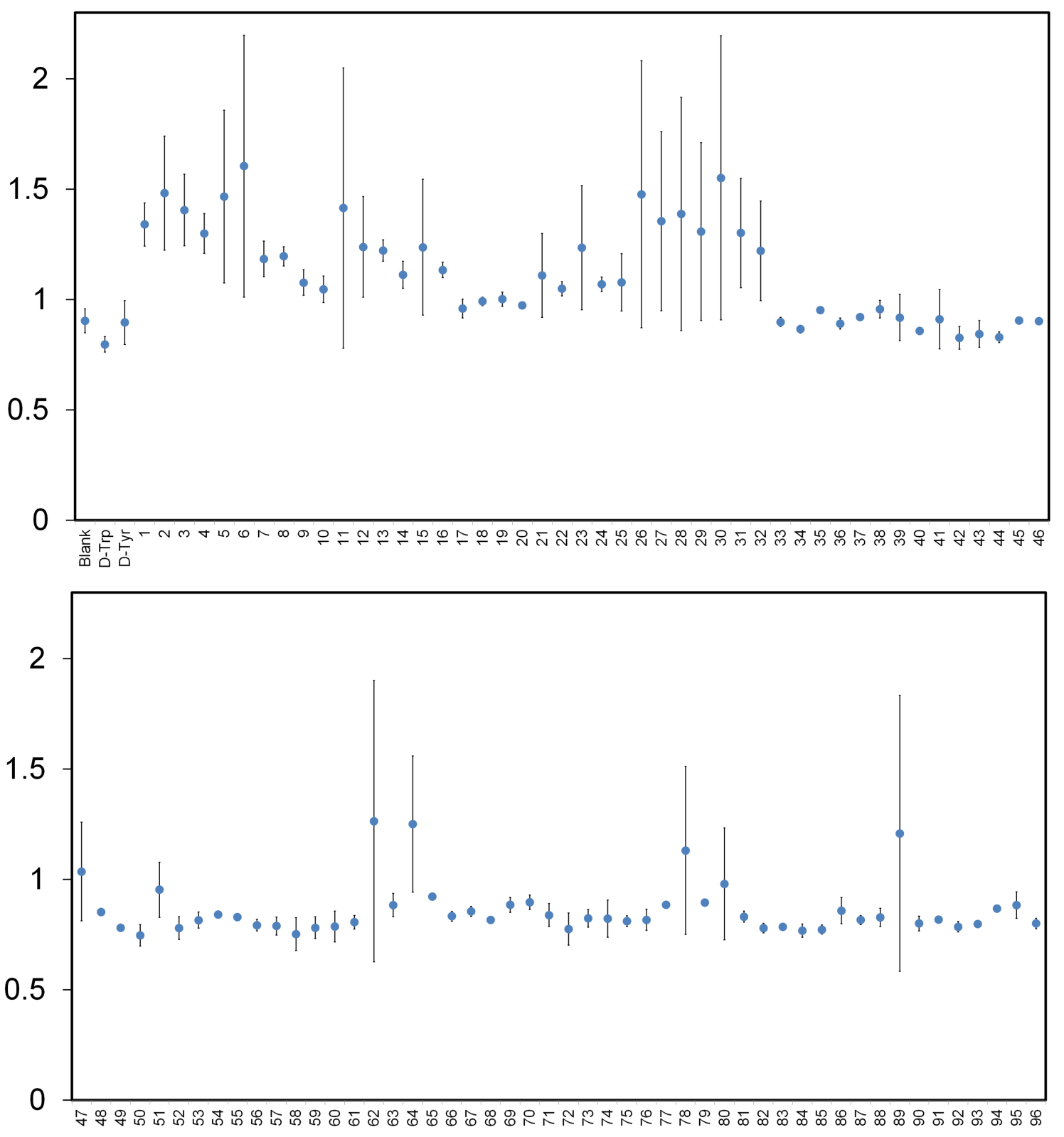


**Figure E.** *S. epidermis* (ATCC 12228) biofilm formation was evaluated after 48 h in the presence of specified D-amino acids at a concentration of 1 mM. The absorbance was recorded at 595 nm following crystal violet staining.


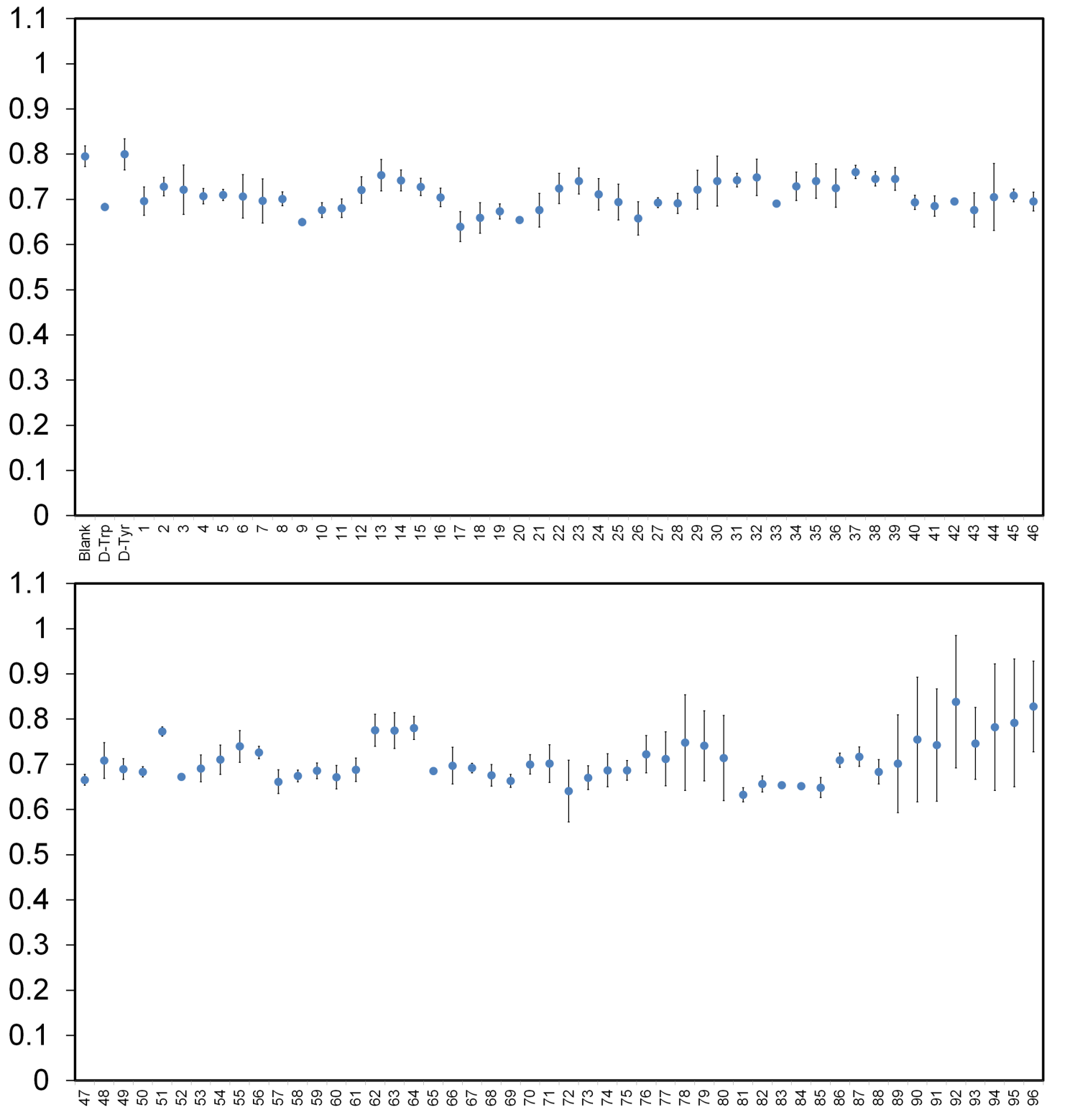


**Figure F.** *B. subtilis* (3610 strain) biofilm formation was evaluated after 3 d in the presence of controls (A) and specified D-amino acids from our panel at a concentration of 1 mM.


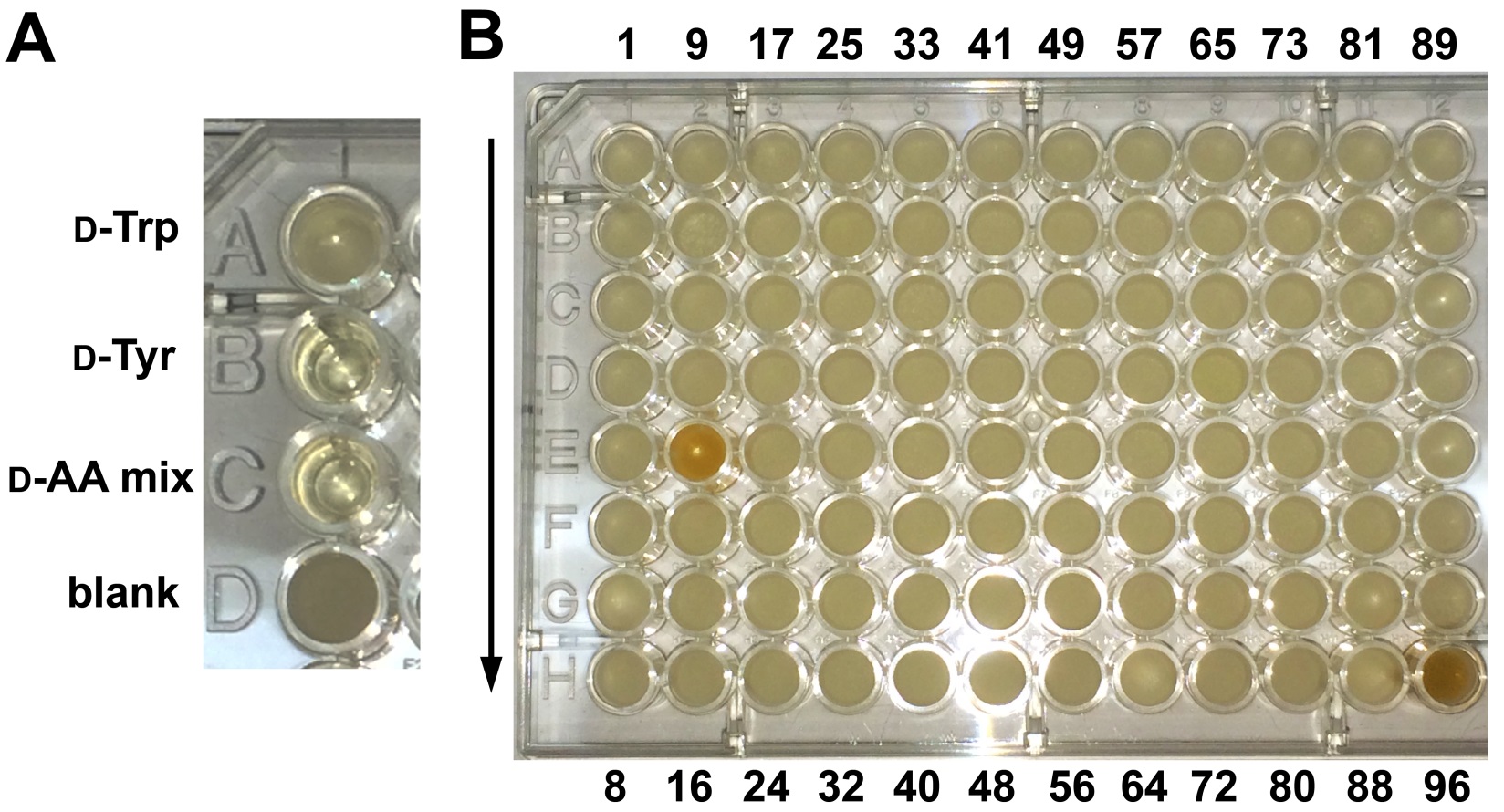

Supplement: S1 File — Table A, Structures of unnatural D-amino acids evaluated. The full structure of the diverse set of D-amino acids evaluated are shown below. Fig. A, S. aureus (SC01) biofilm formation was evaluated after 24 h in the presence of specified D-amino acids at a concentration of 1 mM. The absorbance was recorded at 595 nm following crystal violet staining. Fig. B, S. aureus (SC01) biofilm formation was evaluated after 48 h in the presence of specified D-amino acids at a concentration of 1 mM. The absorbance was recorded at 595 nm following crystal violet staining. Fig. C, S. aureus (SC01) biofilm formation was evaluated after 24 h in the absence of cells, in the presence of cells, and in the presence of the positive control carvacrol (2 mM). The images were taken following crystal violet staining. Fig. D, S. aureus (ATCC 25923) biofilm formation was evaluated after 48 h in the presence of specified D-amino acids at a concentration of 1 mM. The absorbance was recorded at 595 nm following crystal violet staining. Fig. E, S. epidermis (ATCC 12228) biofilm formation was evaluated after 48 h in the presence of specified D-amino acids at a concentration of 1 mM. The absorbance was recorded at 595 nm following crystal violet staining. Fig. F, B. subtilis (3610 strain) biofilm formation was evaluated after 3 d in the presence of controls (A) and specified D-amino acids from our panel at a concentration of 1 mM. (DOCX) [file pone.0117613.s001.docx]
